# Co-Circulation of Tick-Borne *Bandaviruses* and *Orthonairoviruses* Across Humans, Livestock, and Rats in Pakistan: Serologic Evidence and Public Health Implications

**DOI:** 10.3390/v17121620

**Published:** 2025-12-15

**Authors:** Muhammad Ammar, Shengyao Chen, Muhammad Saqib, Jingyuan Zhang, Awais-Ur-Rahman Sial, Asad Zia, Yaohui Fang, Muhammad Khalid Mansoor, Abulimiti Moming, Asim Shahzad, Rehman Hafeez, Aneela Javed, Ali Hassan, Ben Hu, Ali Zohaib, Shu Shen, Fei Deng

**Affiliations:** 1 State Key Laboratory of Virology and Biosafety and National Virus Resource Center, Wuhan Institute of Virology, Chinese Academy of Sciences, Wuhan 430071, China; 2 University of Chinese Academy of Sciences, Beijing 100049, China; 3 Department of Clinical Medicine and Surgery, Faculty of Veterinary Science, University of Agriculture, Faisalabad 38040, Pakistan; 4 Department of Clinical Studies, Faculty of Veterinary & Animal Sciences, Pir Mehr Ali Shah Arid Agriculture University, Rawalpindi 46300, Pakistan; 5 Public Health Reference Laboratory-KP, Peshawar 25100, Pakistan; 6 Department of Microbiology, Faculty of Veterinary & Animal Sciences, The Islamia University of Bahawalpur, Bahawalpur 63100, Pakistan; 7 Center for Disease Control and Prevention of Xinjiang Uygur Autonomous Region, Xinjiang Key Laboratory of Vector-Borne Infectious Diseases, Urumqi 830002, China; 8 Department of Pathology, Faculty of Veterinary & Animal Sciences, The Islamia University of Bahawalpur, Bahawalpur 63100, Pakistan; 9 Department of Parasitology, Faculty of Veterinary & Animal Sciences, The Islamia University of Bahawalpur, Bahawalpur 63100, Pakistan; 10 Department of Biomedicine, Atta-ur-Rahman School of Applied Biosciences (ASAB), National University of Sciences and Technology (NUST), H-12 Campus, Islamabad 44000, Pakistan; 11 Infectious Disease Research Department, King Abdullah International Medical Research Center, P.O. Box 9515, Jeddah 21423, Saudi Arabia; 12 King Saud bin Abdulaziz University for Health Sciences, P.O. Box 9515, Jeddah 21423, Saudi Arabia; 13 Ministry of the National Guard—Health Affairs, P.O. Box 9515, Jeddah 21423, Saudi Arabia

**Keywords:** tick-borne viruses, seroprevalence, neutralizing antibodies, Pakistan, virus circulation

## Abstract

Tick-borne viruses (TBVs) pose significant public health and economic threats. Pakistan has endemic Crimean-Congo hemorrhagic fever virus (CCHFV), but evidence suggests broader TBV circulation. This study assessed the seroprevalence of thirteen TBVs (seven are members of the genus Orthonairovirus and six are members of the genus Bandavirus) in humans, livestock, and rats in Punjab, Pakistan. Serum samples (n = 794: 321 livestock, 253 human, and 220 rat) were collected from the Narowal, Lahore, and Faisalabad districts. Antibodies to viral NPs were detected using the luciferase immunoprecipitation system (LIPS). The overall seroprevalence was 19.14% (152/794); it was highest in livestock (27.10%), then humans (20.55%), and then rats (5.91%). The highest seroprevalence rates were 3.12% for CCHFV in livestock, 3.56% for Yezo virus (YEZV) in humans, and 0.91% for Tamdy virus (TAMV) and Tacheng tick virus 1 (TcTV-1) in rats. Neutralizing antibodies were detected against CCHFV (1 cattle, 4 humans), Bhanja virus (BHAV) (3 livestock, 1 rat), TAMV (1 cattle), Guertu virus (GTV) (1 cattle), and *Dabie bandavirus* (2 cattle). Sixteen samples showed antibodies to both *orthonairoviruses* and *bandaviruses*, indicating co-exposure. Further analysis showed that seropositivity was not randomly distributed. Livestock kept in commercial farming systems and people working mainly outdoors had distinctly higher exposure to TBVs than subsistence livestock and indoor workers. The results supported the circulation of TBVs among hosts within the close socio-economic/ecological integration area of Pakistan. These findings confirm the circulation of CCHFV, SFTSV, GTV, and TAMV; provide the first serologic evidence of BHAV in Pakistan; and underscore the need for further investigation into the potential circulation of additional TBVs. All results demonstrated that multiple TBVs have been circulating among humans, livestock, and rodents in Pakistan.

## 1. Introduction

Tick-borne viral diseases (TBVDs), caused by tick-borne viruses (TBVs), have attracted increasing attention due to the growing threats they pose to global public health and their consequent heavy economic burden on the medical and livestock industries [1,2]. For an extended period of time, the tick-borne CCHFV has been circulating in Pakistan, while serologic evidence suggests the presence of other TBVs having caused human exposure there [3,4,5].

Ticks serve as a critical reservoir of TBVs and act as the major vector for the transmission of TBVs by taking blood meals from hosts. Currently, over 40 tick species from *Hyalomma*, *Haemaphesalis*, and *Rhipicephalus* genera are widely distributed in Pakistan, particularly in the Punjab region, where they frequently infest livestock, including cattle, buffalo, sheep, and goats, and are established as the principal vectors of TBVs [6,7]. Surveys carried out in Punjab have also reported high levels of tick infestation in livestock across several districts in central and northeastern parts of the province, such as Narowal, Lahore and Faisalabad, which share similar ecological conditions [2,8]. Ticks feed as larvae and nymphs on a wide range of vertebrate hosts before developing into adults, which brings them into regular contact with large and small ruminants. Consequently, livestock like cattle, buffalo, goats, and sheep in Punjab could be repeatedly exposed to feeding ticks when moving through farms, grazing areas, and animal markets [9,10]. Humans are at potential risk of exposure when they care for or handle these animals, particularly during herding, milking, slaughtering, and agricultural work, where ticks can easily attach to clothing or exposed skin [11]. Although livestock are the primary hosts, rodents, including rats, can also interact with questing ticks in animal sheds, fodder piles, and grain stores in mixed farm environments, and rodents are recognized as important in maintaining and amplifying several TBV hosts [12]. All this establishes an ecological overlap between humans, livestock, and rats potentially linked by ticks, which is supported by previous field surveys showing high infestation rates of ticks on these animals across Punjab [9,10]. Furthermore, viruses persisting in tick populations throughout generations due to transstadial and transovarial transmission increase their chances of spreading to hosts during the whole life cycle [13].

In Pakistan’s complex tick-borne viral landscape, *bandaviruses* and *orthonairoviruses* represent the most significant public health challenges due to their notable transmissibility and high fatality rates. The *orthonairovirus* CCHFV is one of the most lethal tick-borne pathogens long endemic to Pakistan. Human infection with CCHFV leads to severe hemorrhagic disease with high mortality rates and can trigger nosocomial transmission through human-to-human contact, posing an ongoing threat to livestock workers and healthcare personnel [14]. Among *bandaviruses*, the SFTSV exemplifies an emerging tick-borne viral threat. First identified in China’s Henan Province in 2011 [15], SFTSV has subsequently been reported in South Korea and Japan [16,17], demonstrating a trend of continuous geographical expansion. Seropositivity detected in the Pakistani population in 2020 further indicates the potential risk of local circulation [4]. Although SFTSV shares some clinical manifestations with CCHFV, it is more prone to underdiagnosis, and its true prevalence in Pakistan remains unclear. Research on these viruses is critically important for establishing disease early-warning systems and presents a proactive strategy for mitigating large-scale outbreaks of emerging and re-emerging infectious diseases.

To address this gap, serum samples from humans, livestock, and rats were collected from Punjab’s integrated economic zones, where human–livestock–rodent contact intensifies spillover risk [4,14,18]. Serologic exposure to thirteen TBVs—including seven members of *orthonairoviruses* (TAMV, CCHFV, Wenzhou tick virus (WzTV), Songling virus (SGLV), Huangpi tick virus-1 (HpTV-1), TcTV-1, and YEZV) and six members of *bandaviruses* (SFTSV, GTV, Heartland virus (HRTV), BHAV, Lone Star virus (LSV), and Hunter Island group virus (HIGV))—were investigated, and subsequently neutralization antibodies to specific viruses were examined. Possible routes for TBV circulation among humans, livestock, and rats in Pakistan and potential risk factors were discussed based on the results of serologic tests. The findings will improve our understanding of TBV prevalence and may facilitate the prevention and control of TBV-associated diseases in Pakistan [4,14].

## 2. Materials and Methods

### 2.1. Sample Collection

Serum samples were collected in 2022 from humans, rats, and animals (n = 794: 321 livestock (90 buffalo, 200 cattle, 14 dogs, 12 goats, and 5 sheep), 253 humans (173 males and 80 females), and 220 rats (77 males and 143 females)) in Punjab’s integrated economic zones, which include Narowal (32° N, 74.8° E), Lahore (31.5° N, 74.3° E), and Faisalabad (31.5° N, 74° E). Human sampling employed a convenience strategy due to sociopolitical constraints, limited healthcare infrastructure, and logistical challenges in obtaining farmer participation. Random selection was prioritized where feasible. All the participants gave their informed consent verbally or in writing, regardless of their reading level. Blood (4 mL) was taken from consenting study participants from the peripheral vein into the gel-clot activator containing vacutainer (Improve Medical, Guangzhou, China). Livestock were sampled from households, animal selling markets, and farms where human sampling was conducted, forming spatial correlation for a comparative investigation into exposure to tick-borne viruses. The selection of livestock was also made conveniently, taking into consideration owner permission and farm access.

Rat samples were collected from places where there is high human and animal interaction, particularly from local godowns, shops, livestock farms, and open markets, important sites for the inter-district transportation of food, animals, animal products, crops, and forages. Livestock and rat blood (4 mL) were collected by veterinarians via the jugular or tail veins using identical vacutainers. The samples were shipped to The Islamia University of Bahawalpur, Punjab, Pakistan, under continuous cold-chain maintenance. After centrifugation (5000× *g*, 12 min), serum was aliquoted into cryovials (Imec Medical Co., Zhangjiagang, China) and stored at −40 °C until subjected to further experimentation.

All the procedures involving human participants and animals were reviewed and approved by the Departmental Bioethics Committee, Department of Microbiology, The Islamia University of Bahawalpur (REC. BEC No. 05-2021-21/2; approval date: 2 May 2021), Pakistan, and by the ethics committees of Wuhan Institute of Virology, Chinese Academy of Sciences (WIVHF33202404; approval date: 29 September 2024). These committees also serve as the institutional animal ethics and animal care and use bodies for this project, and all livestock and rat sampling was conducted in accordance with their approved protocols and relevant animal welfare regulations.

### 2.2. Cells, Viruses, and Antibodies

Human embryonic kidney cells (CRL-11268), African green monkey kidney cells (CCL-81), and African green monkey kidney E6 cells (ATCC-1586) were acquired from the American Type Culture Collection (ATCC, Manassas, VA, USA) and cultivated in Eagle’s Minimum Essential Medium (EMBM) (NZK Biotech, Nanjing, China) with 10% FBS and Dulbecco’s Modified Eagle Medium (DMEM) (NZK Biotech, Nanjing, China) with 10% fetal bovine serum (FBS) (Gibco, Grand Island, NY, USA) and 1% penicillin-streptomycin (PS; Gibco, Grand Island, NY, USA).

The SFTSV strain WCH (IVCAS6.6088; GenBank accession no. JQ341190.1), CCHFV strain YL16070 (IVCAS6.6329; GenBank accession no. KY354080.1), TAMV strain YL16082 (IVCAS6.7499; GenBank accession no. MT815991.1), HRTV strain MO-4 (IVCAS6.6330; GenBank accession no.LC629153.1), GTV strain DXM (IVCAS6.6106; GenBank accession no.KT328591.1), HpTV-1 strain HTV1/SZYD9 (IVCAS6.9465; GenBank accession no.MW721869.1), and BHAV strain M3811 (IVCAS6.9001; GenBank accession no. JQ956378.1) were obtained from the National Virus Resource Center (NVRC), China.

Rabbit polyclonal antibodies against nucleoprotein (NP) of SFTSV [19], CCHFV [20], TAMV [21], and GTV [22] were previously prepared, and the rabbit polyclonal antibodies against NPs of HRTV, HpTV-1, and BHAV were prepared in-house following the procedure outlined in previous studies [20,21,22]. The anti-FLAG Tag antibody (Sangon Biotech, Shanghai, China) was used as the primary antibody in verifying the expression of the LIPS-related fusion protein. GAPDH mouse mAb (abclonal, Wuhan, China) was used as the inner control. The goat anti-rabbit IgG (H + L) conjugated with horseradish peroxidase (HRP) (Proteintech, Wuhan, China) was used as the secondary antibody to verify the expression of LIPS-related fusion protein. Goat anti-rabbit IgG H&L conjugated with Alexa Fluor 488 (Abcam, Shanghai, China), goat anti-human IgG H&L conjugated with horseradish peroxidase (HRP) (Abcam, Shanghai, China), and HRP-Protein A (proteintech, Wuhan, China) were used as secondary antibodies for immunofluorescence assays (IFAs) or Western blot.

### 2.3. Reagents

Hoechst 33258 (Beyotime, Shanghai, China) was used for staining cell nuclei. ClonExpress^®^Ultra One-step Cloning Kit (Vazyme, Nanjing, China) for gene cloning in pREN2 plasmids in-frame with the Renilla luciferase (Rluc) and Flag tag. Lipofectamine^TM^ 3000 (Invitrogen, Carlsbad, CA, USA) was used to transfect plasmids into 293T cells. Sea kidney luciferase reporter gene cell lysate buffer (Beyotime, Shanghai, China). Pierce Protein A/G UltraLink Resin beads (Thermo Fisher Scientific, Waltham, MA, USA), Renilla-Lumi™ Plus and Luciferase Assay Kit (Beyotime, Shanghai, China).

### 2.4. Serological Testing Using Luciferase Immunoprecipitation System (LIPS) Followed by Western Blot and Microneutralization Assays

The presence of antibodies was initially detected using the luciferase immunoprecipitation system (LIPS) assays and subsequently confirmed by Western blot as previously described [23]. To construct the plasmids expressing viral antigen, the NP genes of thirteen viruses, including TcTV-1, HpTV-1, WzTV, SGLV, YEZV, HRTV, HIGV, BHAV, and LSV, were cloned into pREN2 plasmids in-frame with the Renilla luciferase (Rluc) and Flag tag by using the ClonExpress^®^Ultra One-Step Cloning Kit (Vazyme, Nanjing, China) according to the manufacturer’s instructions, and were further verified by Sanger sequencing. For CCHFV, TAMV, SFTSV, and GTV, NP-based plasmid constructs and related assays had been previously established and validated by [23] and were used in this study as controls for validating viral protein expression. Subsequently, the plasmids inserted with or without the viral NP gene were transfected into HEK293T cells using Lipofectamine™ 3000 Transfection Reagent (Invitrogen, America). At 48 h post-transfection, cells were fixed to visualize viral protein expression by IFAs or lysed using sea kidney luciferase reporter gene lysis buffer (RG129M, Beyotime, Shanghai, China) and subjected to Western blot to validate expression of viral antigens. The anti-FLAG antibody was used as the primary antibody for both tests. The remaining cell lysates were either stored at −80 °C for later analysis or used immediately for LIPS assays as previously described [23]. The Luminescence Unit (LU) values for each tested sample were measured using Renilla-Lumi™ Plus Luciferase Assay Kit (Beyotime, Shanghai, China) by using a luminometer, the GloMax Multi+ Detection System (Promega, Madison, WI, USA). The cut-off value for antibody positivity was determined by calculating the average and standard deviation (STDEV) of LU values from all tested samples, which was expressed as:

Cut-off = Mean LU + 3 × STDEV

Samples with LU values above this cut-off were considered seropositive.

The expression of the NP genes of thirteen viruses, including CCHFV, TAMV, TcTV-1, HpTV-1, WzTV, SGLV, YEZV, SFTSV, GTV, HRTV, HIGV, BHAV, and LSV, in fusion with Flag-tag in the plasmid-transfected cells was verified by Western blot using anti-FLAG Tag antibody (Sangon Biotech, Shanghai, China) (1:1000 dilution) as the primary antibody and goat anti-rabbit IgG (H + L) conjugated with horseradish peroxidase (HRP) (Proteintech, Wuhan, China) (1:3000 dilution) as the secondary antibody. GAPDH expression was blotted using GAPDH Mouse mAb (abclonal, China) as the inner control. To confirm the seropositive samples identified by LIPS, Western blot was performed using purified respective viral particles as antigens, followed by incubation with serum samples or specific polyclonal viral antibodies as controls and the commercially provided HRP-labeled secondary antibodies specific to animals or humans [23].

To identify neutralizing antibodies, serum samples were heat-inactivated at 56 °C for 30 min to inactivate complement and other non-specific antiviral factors. Then, the presence of neutralizing antibodies against CCHFV, TAMV, HpTV-1, SFTSV, GTV, HRTV, and BHAV were detected using the method as previously described [3,21,22]. IFAs were performed to visualize virus infection in each well. For each dilution, the tests were performed in triplicate. The neutralization titer was defined as the reciprocal of the highest serum dilution that completely prevented virus infection in all three replicate wells.

### 2.5. Infection Assays and Virus Purification

We cultured cells for virus propagation using Vero cells for SFTSV, HRTV, GTV, and BHAV and Vero E6 cells for CCHFV, TAMV, and HpTV-1. Cells were seeded in T75 tissue culture flasks and maintained in Dulbecco’s Modified Eagle Medium (DMEM, 2%) and Eagle’s Minimum Essential Medium (EMEM, 2%), respectively. Both media were supplemented with 10% fetal bovine serum (FBS) and 1% penicillin–streptomycin. Cells were incubated at 37 °C in a humidified atmosphere containing 5% CO_2-_ until they reached approximately 80–90% confluency. For viral infection, 100 µL of virus stock solution was added to each flask, followed by incubation for five days.

At 5 days post-infection (p.i.), supernatants were collected and centrifuged at 4000× *g* to remove residual cells. Viral particles were concentrated using a polyethylene glycol (PEG8000) precipitation method. A 5× PEG8000/NaCl stock solution was prepared by dissolving 50 g of PEG8000 and 8.766 g of NaCl in 200 mL of distilled water and was autoclaved at 121 °C for 30 min. Virus-containing supernatants were filtered through a 0.45 μm membrane filter, mixed with the pre-cold PEG8000/NaCl solution (*w*/*w* = 5:1), and incubated at 4 °C overnight. Viral particles were pelleted by centrifugation at 4000× *g* for 20 min at 4 °C. The viral pellet was harvested and applied to Western blot as antigens to examine antibody response among serum samples [24]. The laboratory-prepared rabbit anti-nucleoprotein (Np) antibodies (1:2000) to respective viruses were used as the primary antibodies. For human serum samples HRP-conjugated goat anti-human IgG (H + L) (1:5000 dilution) or HRP-Protein A (1:5000 dilution) were used as the secondary antibodies to immunoblot antibody response from human serum samples or livestock and rats, respectively.

### 2.6. Phylogenetic Analysis and Pairwise Sequence Identity

The nucleotide sequences of the nucleoprotein (NP) gene segment were aligned by ClustalW (v2.1) implemented in MEGA 11 (version 11.0.10; Mega Limited, Auckland, New Zealand), and the phylogenetic tree was constructed using the maximum likelihood method with 1000 bootstrap replicates. The Gn and NP amino acid sequences were aligned using Clustal Omega from the European Molecular Biology Laboratory-European Bioinformatics Institute (EMBL-EBI, Hinxton, UK; https://www.ebi.ac.uk/Tools/msa/clustalo/, accessed on 15 March 2025) and then analyzed using an online Chiplot tool (https://www.chiplot.online/, accessed on 15 March 2025) to generate the pairwise percent identity matrix. Genotypes were distinguished according to the procedure described in previous studies [25,26].

### 2.7. Statistical Analysis

Statistical analysis was performed using SPSS Statistics Version 21 (IBM Corporation, Armonk, NY, USA) and GraphPad Prism 8 (GraphPad Software, San Diego, CA, USA). Chi-squared testing examined correlations between host risk factors and viral infection with a 95% confidence interval. Positive samples were also analyzed statistically for associations between age, gender, job, and animal contact for humans; sex and environment for rats; animal type (buffalo, cattle, dogs, goats, and sheep), sex, and farming system for livestock by using the χ^2^ test (or Fisher’s exact test) at a 95% confidence interval. For all analyses, a *p*-value of <0.05 was considered significant.

## 3. Results

We collected 794 serum samples from 321 livestock (buffalo, cattle, dogs, goats, and sheep), 253 humans (convenience-sampled), and 220 rats. Antibody responses to seven members of orthonairoviruses (TAMV, CCHFV, WzTV, SGLV, HpTV-1, TcTV-1, and YEZV) and six members of bandaviruses (SFTSV, GTV, HRTV, BHAV, LSV, and HIGV) were examined among these samples by LIPS, as these viruses are either known human pathogens or have potential to infect humans (Supplementary Table S1). Consequently, an expanded LIPS assay was built by including the nine additional TBVs upon the previously established methods for TAMV, CCHFV, SFTSV, and GTV [5]. NP expression of all thirteen TBVs was confirmed in the transfected cells, with those of TAMV, CCHFV, SFTSV, and GTV revalidated as controls (Supplementary Figure S1). Subsequently, the LIPS assay identified an overall seroprevalence rate of 19.14% (152/794), with livestock exhibiting the highest rate (87/321, 27.10%), followed by humans (52/253, 20.55%) and rats (13/220, 5.91%) (Table 1).

Species analysis revealed CCHFV as the most prevalent virus in livestock (10/321, 3.12%), while GTV was the least (4/321, 1.25%). Humans exhibited the highest seroprevalence for Yezo virus (YEZV) (9/253, 3.56%) and the lowest for Tacheng tick virus-1 (TcTV-1) (1/253, 0.40%). Rats demonstrated a higher exposure to TAMV and TcTV-1 (2/220, 0.91%) and lower exposure to the other tested viruses (1/220, 0.45%) (Table 1, Supplementary Figure S2). Antibody responses detected by LIPS for SFTSV, GTV, BHAV, HRTV, TAMV, CCHFV, and HpTV-1 (viruses preserved by China’s NVRC) were validated by Western blot using purified viral particles on 85 LIPS-positive samples. The confirmation rates were 50% for SFTSV (6/12), 40% for GTV (4/10), 36.36% for BHAV (4/11), 75% for HRTV (9/12), 100% for TAMV (11/11), 62.50% for CCHFV (10/16), and 46.15% for HpTV-1 (6/12) (Supplementary Figure S3). The variation in confirmation rates may be affected by the differences in method sensitivity or the preparations of purified viral particles.

Limited serologic cross-reaction within the viral groups was suggested by the amino acid identities of either NPs or GPs among the seven orthonairoviruses (NP:44.24–59.96%; GP: 30.91–68.21%) or among the six bandaviruses (NP: 32.27–74.90%; GP: 36.56–71.65%) (Supplementary Figure S4). However, this cross-reaction was still possible, as some samples were detected by Western blot antibody-positive to multiple viruses (Supplementary Figure S3), and the only cross-reaction was previously documented between the SFTSV and GTV bandaviruses [12]. Sixteen samples (11 livestock, 4 human, and 1 rat) showed simultaneous reactivity to both orthonairoviruses and bandaviruses, thereby indicating co-exposure events to the two viral groups (Supplementary Table S2).

We subsequently compared the seroepidemiological rates of the six *bandaviruses* and seven *orthonairoviruses* regarding the different factors among livestock, humans, and rats so as to identify critical factor(s) which could be associated with seroprevalence of specific viruses (Table S3). Overall, SFTSV exhibited seroprevalence rates significantly higher in sheep (1/5, 20.00%; 95% CI: 3.62–62.45%) than cattle (6/200, 3.00%; 95% CI: 1.38–6.39%) and in people performing outdoor jobs (4/128, 3.12%; 95% CI: 1.22–7.76%) than those performing indoor jobs (0/125, 0.00%; 95% CI: 0.00–2.98%). Seroprevalence rates of GTV were found to be significantly higher in males (5/173, 2.89%; 95% CI: 1.24–6.59%) than females (0/180, 0.00%; 95% CI: 0.00–2.09%) among humans. HRTV revealed significant variation across animal species, with sheep having the highest occurrence (1/5, 20.00%; 95% CI: 3.62–62.45%), followed by goats (1/12, 8.33%; 95% CI: 1.49–35.39%), dogs (1/14, 7.14%; 95% CI: 1.27–31.47%), cattle (4/200, 2.00%; 95% CI: 0.78–5.03%), and buffalo (1/90, 1.11%; 95% CI: 0.20–6.03%). Among humans, the rates for HRTV were significantly higher in males (3/173, 1.73%; 95% CI: 0.59–4.97%) than females (0/180, 0.00%; 95% CI: 0.00–2.09%). The highest prevalence of HIGV was recorded in sheep (1/5, 20.00%; 95% CI: 3.62–62.45%), followed by goats (1/12, 8.33%; 95% CI: 1.49–35.39%), cattle (4/200, 2.00%; 95% CI: 0.78–5.03%), and buffalo (1/90, 1.11%; 95% CI: 0.20–6.03%), with a significant difference between animal species. Similarly, BHAV also presented the highest rates in sheep (1/5, 20.00%; 95% CI: 3.62–62.45%), followed by goats (2/12, 16.67%; 95% CI: 4.70–44.80%), dogs (1/14, 7.14%; 95% CI: 1.27–31.47%), and cattle (1/200, 0.50%; 95% CI: 0.09–2.78%), with statistically significant differences. Moreover, the seroprevalence rates of BHAV in men (1/95, 1.05%; 95% CI: 0.19–5.72%) were significantly higher than in women (4/226, 1.77%; 95% CI: 0.69–4.46%). For CCHFV, the seroprevalence rates were significantly higher in men (1/95, 1.05%; 95% CI: 0.19–5.72%) than in women (9/226, 3.98%; 95% CI: 2.11–7.39%). Animal species were found significantly associated with TAMV seroprevalence, as the highest rates were recorded in sheep (1/5, 20.00%; 95% CI: 3.62–62.45%), followed by goats (1/12, 8.33%; 95% CI: 1.49–35.39%), dogs (1/14, 7.14%; 95% CI: 1.27–31.47%), buffalo (1/90, 1.11%; 95% CI: 0.20–6.03%), and cattle (2/200, 1.00%; 95% CI: 0.27–3.57%). Similar results were observed for HpTV-1, WzTV, and SGLV, which showed identical rates that were highest among sheep, followed by goats, dogs, cattle, and buffalo, with statistically significant differences. YEZV was found in higher rates in people aged ≤30 (9/121, 7.44%; 95% CI: 3.69–13.53%) than in those aged >30 (0/132, 0.00%; 95% CI: 0.00–2.83%), in men (9/173, 5.20%; 95% CI: 2.76–9.59%) than women (0/180, 0.00%; 95% CI: 0.00–2.09%), and in those working indoors (9/125, 7.20%; 95% CI: 3.83–13.12%) than those working outdoors (0/128, 0.00%; 95% CI: 0.00–2.91%).

Neutralization tests for antibodies specific to the TBVs available in the NVRC (SFTSV, GTV, BHAV, HRTV, TAMV, CCFHV, and HpTV-1) were performed; tests for HIGV, LSV, YEZV, SGLV, TcTV-1, and WzTV were not possible due to the lack of live viruses. The results demonstrated that specific infection occurred in 13 of the 85 LIPS-positive samples, including in eight livestock (8/321, 2.49%), four humans (4/253, 1.58%), and one rat (1/220, 0.45%) (Table 1; Supplementary Table S2). Of those, the livestock (cattle: L143; 1/321, 0.31%) and four people (4/253, 1.58%) exhibited neutralizing activities to CCHFV, as demonstrated by the neutralization antibody titers of 160 in the cattle and 40 in the four individuals. Three livestock (3/321, 0.93%), including two goats (L71 and L195), one sheep (L173), and one rat (R218; 1/220, 0.45%) exhibited neutralizing activities to BHAV, resulting in titers ranging from 20 to 40. One cattle (L29; 1/321, 0.31%) was neutralizing-positive for TAMV with a titer of 20, while one other was for GTV (cattle: L123; 1/321, 0.31%) with a titer of 40. Two livestock showed neutralization to SFTSV (cattle: L246 and L302; 2/321, 0.62%) with titers of 40 (Table S2). These results confirmed the presence of TAMV in Pakistan, supporting earlier findings [5], and revealed livestock reservoirs for SFTSV and GTV beyond known human infections [4,5]. Neutralization to BHAV provided the first evidence of BHAV presence in Pakistan. No cross-neutralization was observed among the 13 samples (Supplementary Table S2).

## 4. Discussion

**Figure 1:**
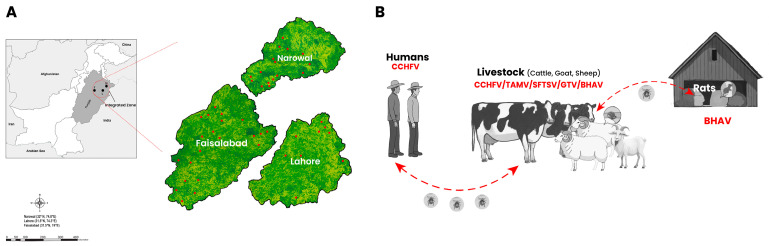
Schematic of sampling sites, human-livestock-rodent interfaces, and circulation of tick-borne viruses (TBVs) in Pakistan. (**A**) A map showing Punjab’s integrated economic zones (indicated by a red dotted circle) where samples were collected in this study. This zone is composed of Narowal (N), Lahore (L), and Faisalabad (F). Zoomed-in district maps display sampling locations as red dots. Faisalabad processes significant livestock, forage, and by-products for domestic and export markets. Narowal supplies key rice and forage to Lahore and Faisalabad, which function as primary storage/distribution centers. Lahore hosts a major provincial livestock market, acting as a central hub for animals sourced locally and across wider regions. It is a highly integrated socio-economic unit with frequent movement of people, animals, and goods. This intense activity occurs in rural/peri-urban areas where rodents (notably rats) inhabit fields, warehouses, and shelters and livestock are reared near human residences, enabling frequent human–animal contact and rodent coexistence. (**B**) The circulation of TBVs among humans, livestock, and rats is based on the seroprevalence observed in this study. Viruses that were identified as being positive for neutralizing antibodies were indicated by red characters in their respective hosts. Red arrows indicate the potential circulation of CCHFV between livestock and humans, and BHAV between livestock and rats according to their identified neutralizing antibodies and possible contacts within the eco-social and ecological surroundings. The figure was created using Adobe Photoshop (Adobe Inc., San Jose, CA, USA) for illustrative purposes.

This study provides serological evidence for the circulation of multiple TBVs among humans, livestock, and rats in the integrated zones of Narowal, Lahore, and Faisalabad in Punjab, where there is a socio-ecological network for the frequent movement of livestock, forage, and animal products that promotes virus dissemination (Figure 1A). Lahore hosts one of the province’s largest livestock markets (mandis), where livestock from not only nearby districts such as Narowal and Faisalabad but also from throughout Punjab and other provinces of Pakistan are transported for the purpose of trade, slaughter, or redistribution [8]. Narowal, a region distinguished by its significant rice and forage production, supplies these to Lahore and Faisalabad, which function as major storage and distribution centers for the wider supply of Punjab and other provinces [8]. Faisalabad, a prominent industrial hub, receives substantial quantities of livestock, forage, and animal by-products. These are processed within the city to satisfy domestic consumption needs and to facilitate export to other regions [8]. These interactions occur in rural and peri-urban regions where rodents such as rats are frequently found in fields, warehouses, and animal shelters, and livestock are grown close to residential areas [18]. In this region, humans, animals, and rodents coexist in close proximity, thereby generating conditions conducive to the circulation of viruses among these populations [18].

This study investigated the seroprevalence of seven members of orthonairoviruses (TAMV, CCHFV, WzTV, SGLV, HpTV-1, TcTV-1, and YEZV) and six members of bandaviruses (SFTSV, GTV, HRTV, BHAV, LSV, and HIGV) by LIPS among livestock, humans, and rats collected from the integrated zones. The LIPS assay is a highly sensitive liquid-phase immunoassay method that has been widely used for high-throughput detection of antibodies against viral proteins and is characterized by its ability to preserve native antigen conformation, producing high sensitivity and low background, which improves sensitivity and specificity, unlike solid-phase assays such as ELISA [22,27]. However, due to its high sensitivity, this method may detect non-specific antibody–antigen reactions, which could be included in the resulting positive rates when multiple antigenically related viruses are tested in parallel. Nevertheless, LIPS could still be considered for the initial screening of a large number of samples from different host species, which could be followed by a different method to verify or confirm the reactions using a different antigen. Therefore, we verified the LIPS-positive samples by Western blot using purified viral particles as antigen. LIPS detects antibodies based on the conformational recognition of viral NPs, while Western blot detects antibodies against linear, denatured epitopes in viral particles. This led to the different sensitivities in the two methods. The number of viral particles used in this study and their differences in antigenicity may also have affected the Western blot analyses confirmation rates. Nevertheless, we identified antibody responses to SFTSV, GTV, BHAV, HRTV, TAMV, CCHFV, and HpTV-1 from the LIPS-positive samples and subsequently confirmed the antibody responses to SFTSV, GTV, BHAV, HRTV, TAMV, CCHFV, and HpTV-1. Neutralizing antibodies specific to SFTSV, GTV, BHAV, and TAMV were further identified. Despite this, there could be false positive or negative outcomes during the initial survey of the serum samples; subsequent examination using different methods would help to confirm specific antibody reactions, particularly by the neutralization assays.

The results by LIPS showed YEZV and CCHFV had the highest seroprevalence rates of all the tested TBVs in the three investigated hosts and the highest subtotal rate of all tested viruses among livestock animals (Table 1). These findings suggest a more comprehensive seroprevalence of the tick-borne orthonairoviruses and bandaviruses among livestock, humans, and rats beyond those reported in the previous study [5]. By analyzing the variations in the positive rates of each virus among livestock, humans, and rats, we found that the livestock species may commonly play a significant role in the seroprevalence of SFTSV, HRTV, HIGV, BHAV, TAMV, TcTV-1, HpTV-1, WzTV, and SGLV, even though the highest rates were not recorded in the same livestock species. Of the livestock species, sheep exhibited the highest rates of up to 20.00% for most of the tested TBVs, probably attributable to the limited sample size of five individuals. Further surveys including more samples from sheep would reveal a more precise seroprevalence rate of these viruses. Furthermore, gender could be another critical factor that affected the seroprevalence of GTV, BHAV, CCHFV, and YEZV among humans. Men generally exhibited significantly higher rates than women, probably due to greater engagement in outdoor occupations such as livestock handling, farming, herding, and animal market work, which place them in frequent contact with tick-infested environments. Consequently, higher rates of SFTSV and YEZV were found among people performing outdoor jobs than those indoors. The prevalence rates for all tested TBVs were not significantly associated with either gender or rat habitat.

Together with the results from the identification of neutralizing antibodies, our data echoes the previous findings of CCHFV prevalence in Pakistan [3,5] and suggests a high likelihood of CCHFV circulation among humans and livestock. The results also indicated the presence and dissemination of BHAV, which is capable of infecting the central nervous system in humans [28], livestock, and rodents for the first time in Pakistan. Since BHAV was identified in ticks in India in 1954 [23], it has been necessary to conduct subsequent surveys on the prevalence of BHAV and its association with human disease in Pakistan. Furthermore, the presence of neutralizing antibodies indicated past infection of SFTSV and GTV in livestock, thereby revealing the expanding SFTSV and GTV seroprevalence not only in humans [4,5]. The presence of TAMV in Pakistan was also confirmed by further serologic evidence that supports the previous findings [5]. Taken together, the identification of neutralizing antibodies against CCHFV in humans and livestock; against TAMV, SFTSV, and GTV in livestock (including cattle, goats, and sheep); and against BHAV in livestock and rats suggests that these TBVs were comprehensively prevalent in Pakistan. Given that the samples were from the integrated zones in Punjab, where human, livestock and rats had frequent contact (Figure 1A), the potential circulating links of CCHFV between humans and livestock and BHAV between livestock and rats were proposed. Moreover, livestock species exhibited neutralizing antibodies to more TBVs, probably because they were more likely to be exposed to the parasite by ticks, resulting in an increased incidence of exposure to TBVs in addition to CCHFV (Figure 1B). These results suggest the need for further investigation of the above TBVs in an expanded region with a larger sample size from hosts as well as ticks.

A key limitation of this study is the inherent sampling bias due to narrow geographic coverage and a non-random sampling approach. Samples were gathered from only three areas within Punjab’s integrated socio-economic zone, which may not completely represent the larger ecological and epidemiological diversity across Pakistan. Human participants were included by convenience sampling due to geopolitical constraints and limited access to rural communities, potentially overrepresenting individuals with higher health-seeking behavior or closer linkages to livestock agricultural activities. Similarly, livestock and rat sampling depended on owner consent, farm accessibility, and the availability of trapping sites, which may have biased detection toward settings with increased human–animal interaction. These restrictions limit the generalizability of the seroprevalence estimates and may either underestimate or overstate the true burden of tick-borne viruses in the region. Future large-scale, active surveillance, including systematic tick sampling and community-based randomized host sampling, will be required to overcome these biases and more precisely determine TBV circulation in Pakistan. Despite these limitations, the circulation of the virus among them was evident as samples were taken from within an ecologic and socio-economic unit. Due to the lack of live viruses, neutralization to HIGV, LSV, YEZV, WzTV, SGLV, and TcTV-1 could not be analyzed. Future research should focus on combining cross-neutralization assays and phylogenetic analysis to strengthen serological specificity, clarify potential cross-reactivity among antigenically related viruses, and improve our understanding of virus–host relationships.

## 5. Conclusions

This study developed LIPS assays for an initial screening of antibodies against seven tick-borne orthonairoviruses and six tick-borne bandaviruses, followed by confirmation using Western blot and microneutralization tests. The results revealed substantial seroprevalence of these viruses in human, livestock, and rodent populations in Punjab, Pakistan, and identified neutralizing antibodies against CCHFV, TAMV, SFTSV, GTV, and BHAV. The findings clearly demonstrate a significant exposure risk to the tick-borne *orthonairoviruses* and *bandaviruses* in Pakistan, which highlights the necessity for implementing an integrated “One Health” monitoring strategy that encompasses ticks, livestock, rodents, and high-risk human groups in this region.

## Figures and Tables

**Table 1:** Seroprevalence rates of thirteen tick-borne viruses across livestock, human, and rat populations in Pakistan based on the results of LIPS and neutralization tests.

Metrics	Livestock (n = 321)		Humans (n = 253)		Rats (n = 220)		Subtotal	
	LIPS Positive	Neutralization Positive/ Rate	LIPS Positive	Neutralization Positive/ Rate	LIPS Positive	Neutralization Positive/ Rate	LIPS Positive	Neutralization Positive/ Rate
TAMV (n, %)	6, 1.87%	1, 0.31%	3, 1.19%	0	2, 0.91%	0	11, 1.39%	1, 0.12%
CCHFV (n, %)	10, 3.12%	1, 0.31%	5, 1.98%	4, 1.58%	1, 0.45%	0	16, 2.01%	5, 0.62%
WzTV (n, %)	7, 2.18%	N/A	2, 0.79%	N/A	0	N/A	9, 1.13%	N/A
YEZV (n, %)	6, 1.87%	N/A	9, 3.56%	N/A	1, 0.45%	N/A	16, 2.02%	N/A
SGLV (n, %)	5, 1.56%	N/A	4, 1.58%	N/A	1, 0.45%	N/A	10, 1.26%	N/A
HpTV-1 (n, %)	7, 2.18%	0	5, 1.98%	0	1, 0.45%	0	13, 1.64%	0
TcTV-1 (n, %)	8, 2.49%	N/A	1, 0.40%	N/A	2, 0.91%	N/A	11, 1.39%	N/A
SFTSV (n, %)	7, 2.18%	2, 0.62%	4, 1.58%	0	1, 0.45%	0	12, 1.51%	2, 0.25%
GTV (n, %)	4, 1.25%	1, 0.31%	5, 1.98%	0	1, 0.45%	0	10, 1.26%	1, 0.12%
HRTV (n, %)	8, 2.49%	0	3, 1.19%	0	1, 0.45%	0	12, 1.51%	0
HIGV (n, %)	7, 2.18%	N/A	3, 1.19%	N/A	1, 0.45%	N/A	11, 1.39%	N/A
BHAV (n, %)	5, 1.56%	3, 0.93%	5, 1.98%	0	1, 0.45%	1, 0.45%	11, 1.39%	4, 0.50%
LSV (n, %)	7, 2.18%	0	3, 1.19%	0	0	0	10, 1.26%	0
Total	87, 27.10%	8, 2.49%	52, 20.55%	4, 1.58%	13, 5.91%	1, 0.45%	152, 19.14%	13, 1.63%

N/A indicates not available, as neutralization tests could not be performed due to the unavailability of virus sources.

## Data Availability

The original contributions presented in this study are included in the article/supplementary material. Further inquiries can be directed to the corresponding author(s).

## Supplementary Materials

The following supporting information can be downloaded at https://www.mdpi.com/article/10.3390/v17121620/s1, Figure S1. Validation of NP protein expression in HEK293T cells transfected with respective plasmids by (A) Western blot analyses and (B) immunofluorescence assays. NC, negative control. Bars, 150 μm; Figure S2. Antibody responses to thirteen tick-borne viruses among livestock (A), humans (B), and rats (C) by using the luciferase immunoprecipitation system. The levels of antibody response to each specific virus were expressed as fold changes to cut-off values; Figure S3. Western blot assay to examine antibody responses to (A) SFTSV, (B) GTV, (C) BHAV, (D) HRTV, (E) TAMV, (F) CCHFV, and (G) HpTV-1. Sample IDs labeled in red indicate the LIPS-positive samples; samples positive with antibody responses to multiple viruses marked with symbol “#”; and samples that were confirmed with neutralization are marked by an asterisk symbol *; Figure S4. Pairwise sequence identity and phylogenetic analysis of (A) Bandaviruses and (B) Orthonairoviruses. The pairwise percent identities of NP (bottom left) and Gn (top right) amino acid sequences aligned with the respective phylogenetic trees, with genotypes indicated. The sequence of Hantavirus was used as an outgroup for both trees; Table S1. Taxonomic and biological characteristics of the seven Orthonairoviruses and six Bandaviruses investigated in this study; Table S2. Background information and detailed results of the cases that possess positive serological activities against tick-borne viruses in livestock, humans, and rats. Table S3. Univariable analysis of associations between host demographic factors and thirteen tick-borne viruses seropositivity in livestock, humans, and rats.
