# Supplementary figures and images for: Co-Circulation of Tick-Borne Bandaviruses and Orthonairoviruses Across Humans, Livestock, and Rats in Pakistan: Serologic Evidence and Public Health Implications
Source: Viruses. 2025 Dec 15;17(12):1620. doi: 10.3390/v17121620 (PMC12737738; doi:10.3390/v17121620)

Figure S1

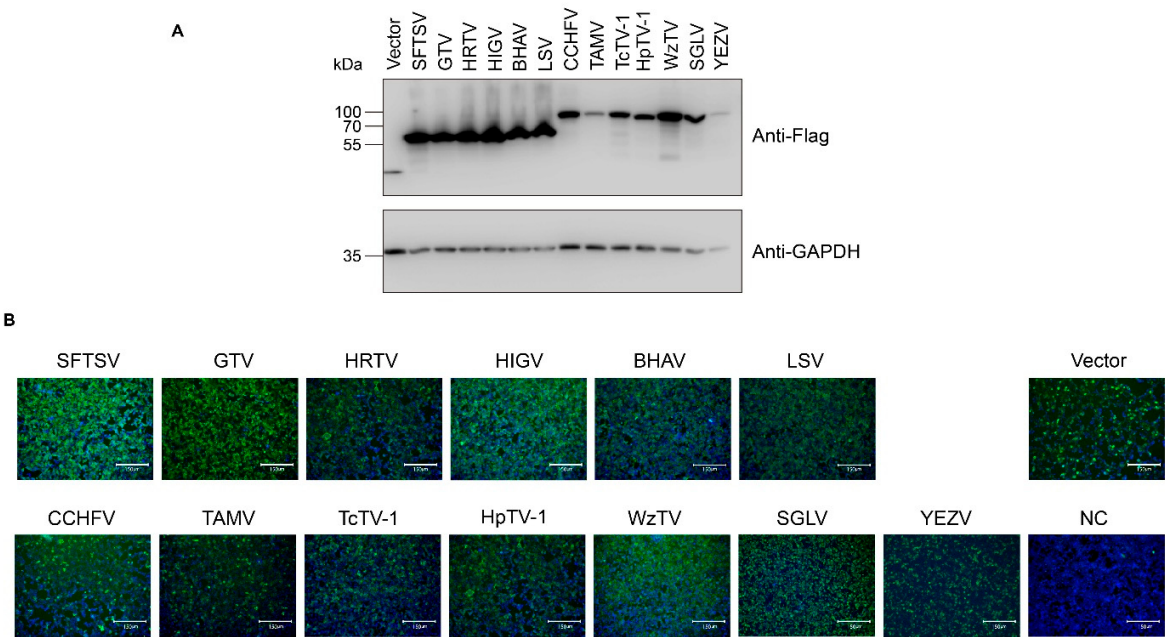

Supplement: Supplementary file 1 [file viruses-17-01620-s001.zip › Supplementary Figure S1.pdf]

Figure S2

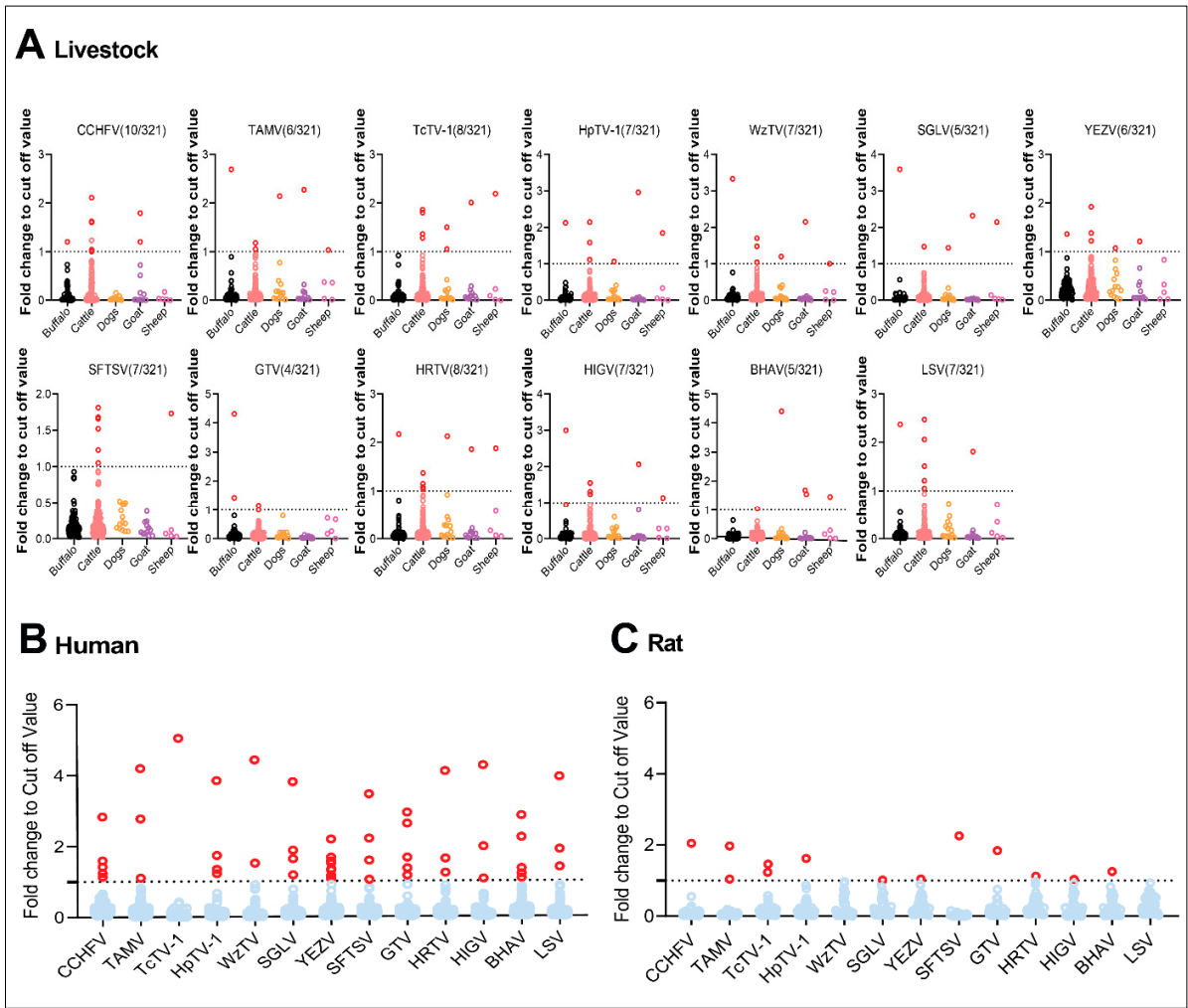

Supplement: Supplementary file 1 [file viruses-17-01620-s001.zip › Supplementary Figure S2.pdf]

Figure S3

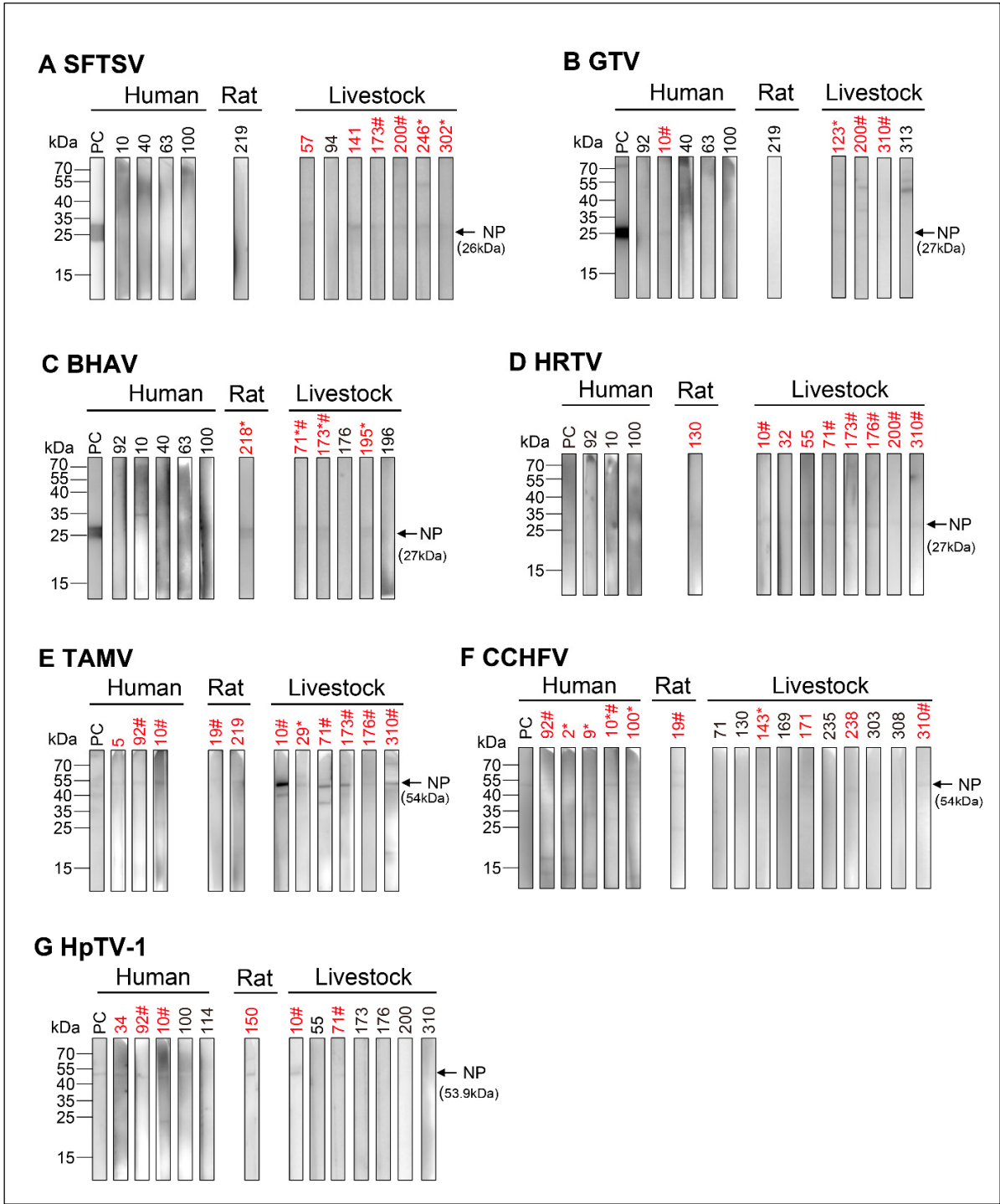

Supplement: Supplementary file 1 [file viruses-17-01620-s001.zip › Supplementary Figure S3.pdf]

Figure S4

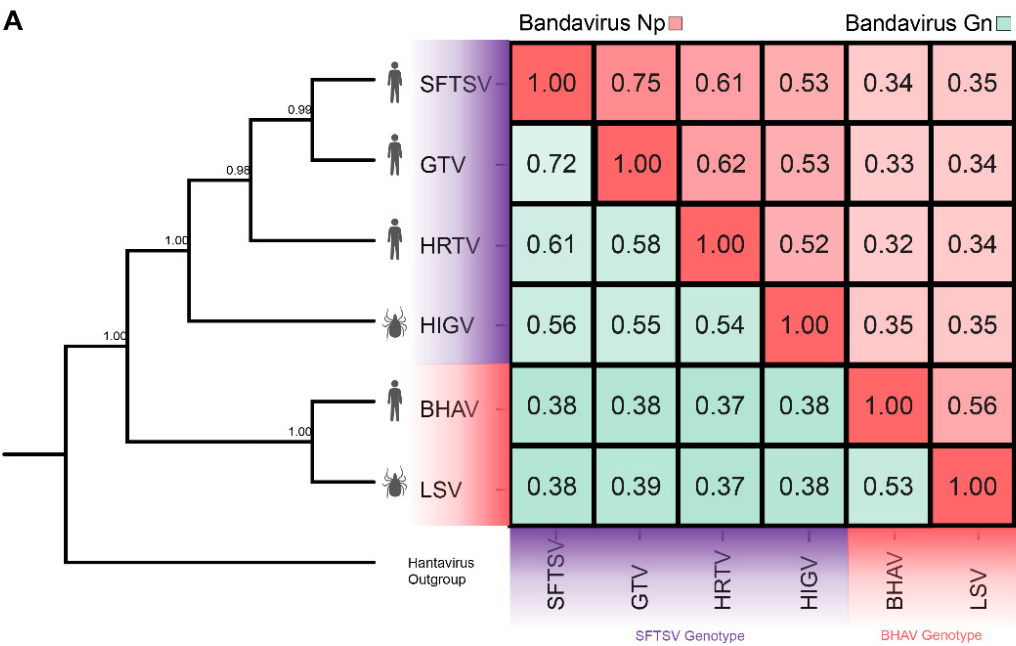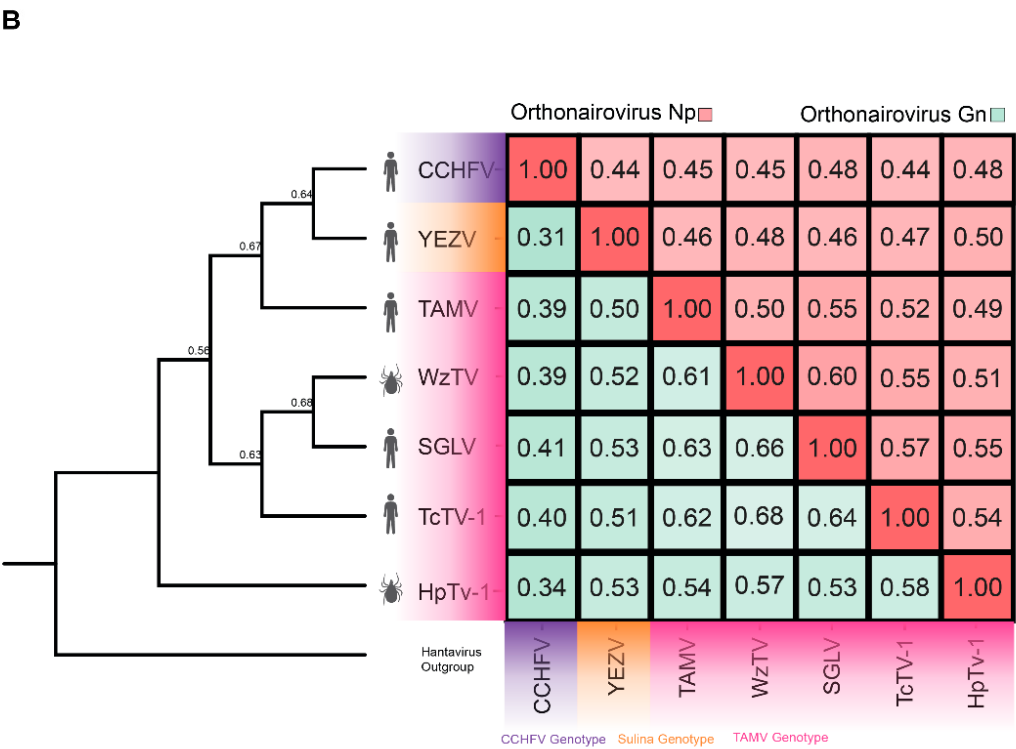

Supplement: Supplementary file 1 [file viruses-17-01620-s001.zip › Supplementary Figure S4.pdf]
